# Open Parenchyma-Preserving Enucleation of Giant Hepatic Hemangiomas: A Systematic Cavitron Ultrasonic Surgical Aspirator (CUSA)-Guided Pringle-Free Technique

**DOI:** 10.7759/cureus.106383

**Published:** 2026-04-03

**Authors:** Mohammad Khalifeh, Joanna Khalifeh, Ahmad Jradi, Riwa Deghaim, Mohamad El Haress, Mustafa Natout, Walid Faraj

**Affiliations:** 1 Department of Surgery, American University of Beirut Medical Center, Beirut, LBN; 2 Reproductive Medicine, Fetal Medicine Research Institute, King's Fertility, London, GBR; 3 Faculty of Medicine and Medical Sciences, University of Balamand, Koura, LBN; 4 Department of Diagnostic Radiology, American University of Beirut Medical Center, Beirut, LBN; 5 Liver and Pancreatic Surgery, Clemenceau Medical Center, Dubai, ARE

**Keywords:** blood loss, cavernous, hemangioma, hepatic hepatectomy ultrasonography, interventional liver neoplasms, parenchyma preserving, pringle maneuver, surgical organ sparing treatments, ultrasonic surgical procedures

## Abstract

Giant hepatic hemangiomas, particularly those involving central hepatic segments (IV, V, and VIII), present unique surgical challenges due to their proximity to major hepatic veins and first-order portal structures. While laparoscopic approaches have been described, detailed guidance for open enucleation across a range of lesion locations and complexities remains limited. This technical report describes a systematic open parenchyma-preserving enucleation technique and presents a retrospective consecutive case series of six patients who underwent the described procedure over an eight-year period at a tertiary hepatobiliary center (American University of Beirut Medical Center, AUBMC). Patient demographics, tumor characteristics, operative details, and postoperative outcomes were retrospectively extracted from medical records following a structured chart review. All patients were followed clinically postoperatively, with no evidence of recurrence or late complications during the available follow-up period. Six patients (5 female, 1 male), with ages ranging from 26 to 50 years, underwent open enucleation using a uniform Cavitron Ultrasonic Surgical Aspirator (CUSA)-guided pseudocapsular technique without inflow occlusion (Pringle maneuver). Tumor locations included purely central segments (n=2), mixed central and peripheral segments (n=3), and a complex suprahepatic lesion with inferior vena cava (IVC) involvement (n=1). Preoperative tumor sizes ranged from 7 to 12.9 cm. Documented estimated blood loss ranged from 150 to 500 mL; five of six cases required no transfusion. One case involving direct IVC adherence required primary suture repair and intraoperative transfusion (3 units; Clavien-Dindo grade III). One patient experienced urinary retention (Clavien-Dindo grade I). No conversions to formal hepatectomy, bile leaks, postoperative liver failure, or mortality occurred. Length of hospital stay ranged from 5 to 10 days. All pathology specimens confirmed cavernous hemangioma. The described CUSA-guided pseudocapsular enucleation technique without the Pringle maneuver is feasible and safe for giant hepatic hemangiomas across central, peripheral, and complex tumor locations. This technical report provides detailed operative guidance applicable across central and peripheral tumor locations, with particular attention to centrally located lesions where proximity to major hepatic veins and portal structures demands structured operative planning and heightened technical vigilance.

## Introduction

Hepatic hemangiomas are the most common benign liver tumors, with an estimated prevalence of 0.4% to 20% in the general population [1]. The majority remain asymptomatic and require no intervention; conservative management with serial imaging surveillance is appropriate for most patients. Non-surgical options, including transcatheter arterial embolization (TAE), have been described for symptomatic lesions in patients who are poor surgical candidates, though surgical enucleation or resection remains the definitive treatment for giant or symptomatic hemangiomas requiring intervention. Giant hepatic hemangiomas have been variably defined in the literature as lesions exceeding 5 to 10 cm in diameter. In this report, we adopt a threshold of 5 cm, consistent with published series emphasizing symptomatic or anatomically complex lesions regardless of absolute size [2,3]. These lesions can become symptomatic through compression of adjacent organs, causing abdominal pain or early satiety, and in some cases, diagnostic uncertainty or significant symptoms may require surgical management.

Hemangiomas involving central hepatic segments IV, V, and VIII present unique anatomical challenges distinct from their peripheral counterparts. They are located in close proximity to major liver structures, including the main portal pedicles, the hepatic hilum, major hepatic veins, and the inferior vena cava (IVC). The compressed parenchyma surrounding these tumors provides limited working space and may compromise visualization, contributing to increased surgical complexity and potential for major vascular or biliary injury [4,5].

Multiple comparative studies and meta-analyses have demonstrated that enucleation is associated with improved perioperative outcomes compared with formal hepatectomy for giant hepatic hemangiomas, including reduced operative time, decreased blood loss, lower transfusion requirements, and diminished postoperative morbidity [6-9]. The theoretical advantage of enucleation derives from its parenchyma-preserving nature, exploiting the pseudocapsule formed by compressed liver tissue surrounding the hemangioma. However, the published literature predominantly addresses peripheral lesions or provides limited operative detail applicable to centrally located tumors [10,11].

Notably, the core operative technique of pseudocapsular enucleation is fundamentally uniform regardless of tumor location. What differs between central and peripheral lesions is not the technique itself, but the degree of anatomical complexity, the risk profile of dissection near major vascular structures, and the critical importance of structured intraoperative decision-making. This technical report describes the procedure as applied across our complete institutional experience while providing particular operative guidance for the more challenging central cases.

Hepatic hemangiomas demonstrate a marked female predominance, with reported female-to-male ratios ranging from approximately 3:1 to 5:1, suggesting a hormonal influence in their pathogenesis [12,13]. Prospective observational data indicate that estrogen exposure, both endogenous and exogenous, may be associated with interval lesion enlargement [12,14]. Consistent with this, two patients in our series had documented hormonal exposure histories: one with prior oral contraceptive use and one with a prior cesarean delivery.

We describe a detailed step-by-step operative approach to open enucleation of giant hepatic hemangiomas based on a retrospective consecutive series of six cases performed at our institution, with particular technical emphasis on centrally located lesions involving segments IV, V, and VIII. This manuscript is intended as a technical educational report with illustrative clinical experience, rather than a comparative or outcomes study. To our knowledge, this report provides the most detailed published operative description of open pseudocapsular enucleation applied across a spectrum of central and complex hepatic hemangioma locations, with explicit intraoperative ultrasound (IOUS)-guided entry point methodology, a Pringle-free strategy justified on navigational rather than purely hemostatic grounds, and structured intraoperative decision criteria for conversion.

## Technical report

Patient selection

**Figure 1:**
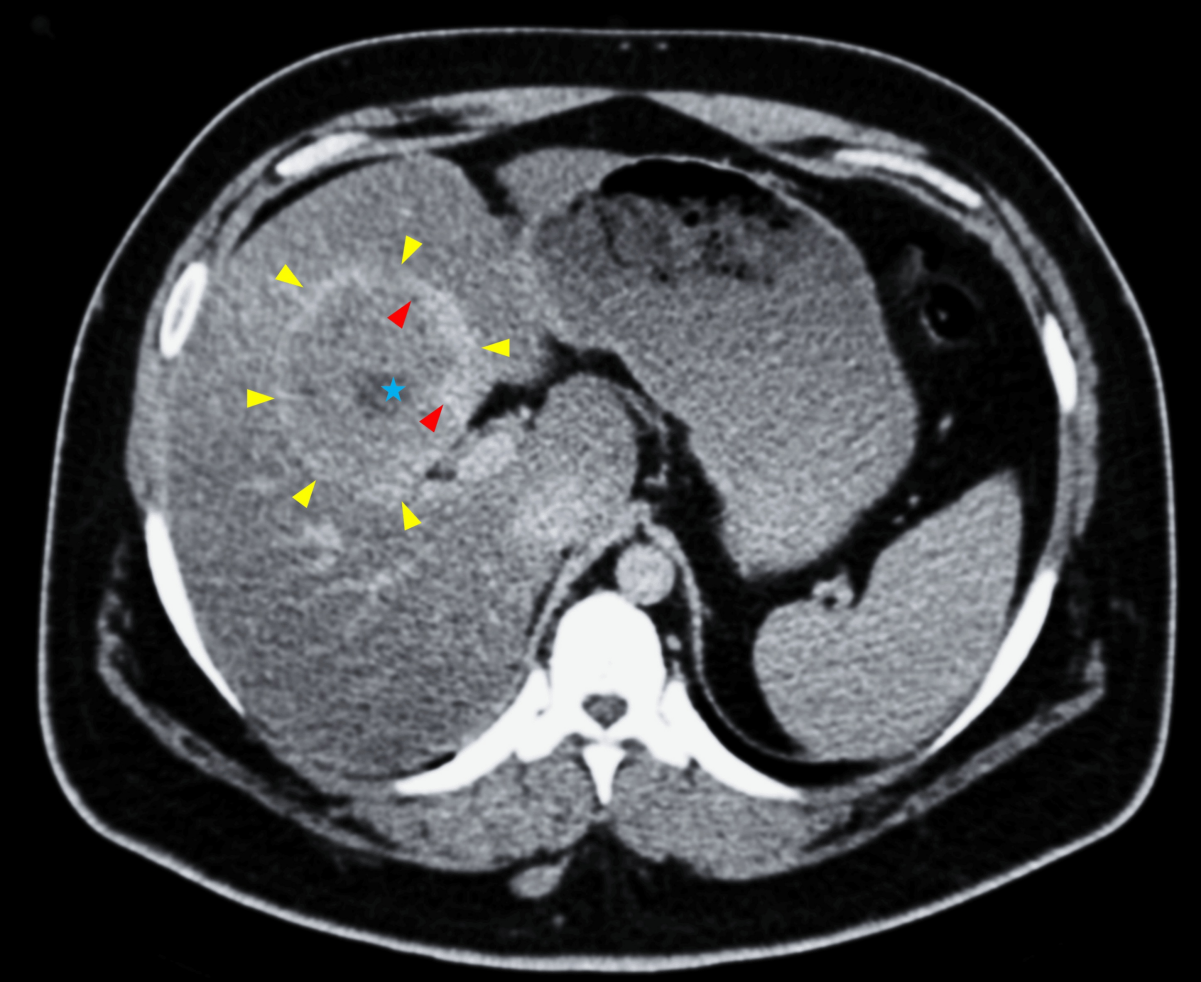
Axial image of intravenous contrast-enhanced CT of the upper abdomen demonstrating a giant cavernous hemangioma (yellow arrowheads) in segment IVb of the liver Interrupted nodular peripheral enhancement (red arrowheads) and a small central scar (blue star) represent atypical imaging features prompting a multidisciplinary review to exclude malignancy.

Surgical intervention was considered for patients with giant hepatic hemangiomas (≥5 cm) when at least one of the following indications was present: (1) persistent symptoms attributable to the lesion, such as right upper quadrant or epigastric pain, early satiety, or compression-related mass effect, persisting despite conservative management and impacting quality of life. Prior to attributing symptoms to the hemangioma, alternative diagnoses were excluded through standard preoperative workup, including cross-sectional imaging and upper gastrointestinal endoscopy, where clinically indicated. Symptoms were considered attributable to the lesion when imaging demonstrated mass effect on adjacent structures and no alternative pathology was identified. (2) progressive enlargement on serial imaging, defined as a clinically meaningful increase in maximal diameter confirmed on at least two cross-sectional imaging studies (CT or MRI) performed 6-12 months apart; (3) diagnostic uncertainty, including the absence of classic hemangioma imaging characteristics or the presence of atypical features, such as non-classic enhancement patterns, including interrupted peripheral enhancement and central scarring (Figure 1), washout-like behavior, restricted diffusion, or discordant imaging findings prompting multidisciplinary review to exclude malignancy. In cases of diagnostic uncertainty, surgical decision-making was guided by multidisciplinary review. Enucleation was selected only after imaging characteristics were deemed sufficiently consistent with hemangioma following this review; intraoperative frozen section was available if needed. All pathology specimens retrospectively confirmed cavernous hemangioma, validating the preoperative assessments; or (4) rare hemangioma-related complications such as consumptive coagulopathy or significant compression of adjacent vascular structures. Lesion size alone, in the absence of symptoms, growth, or diagnostic uncertainty, was not considered an indication for operative management.

**Figure 2:**
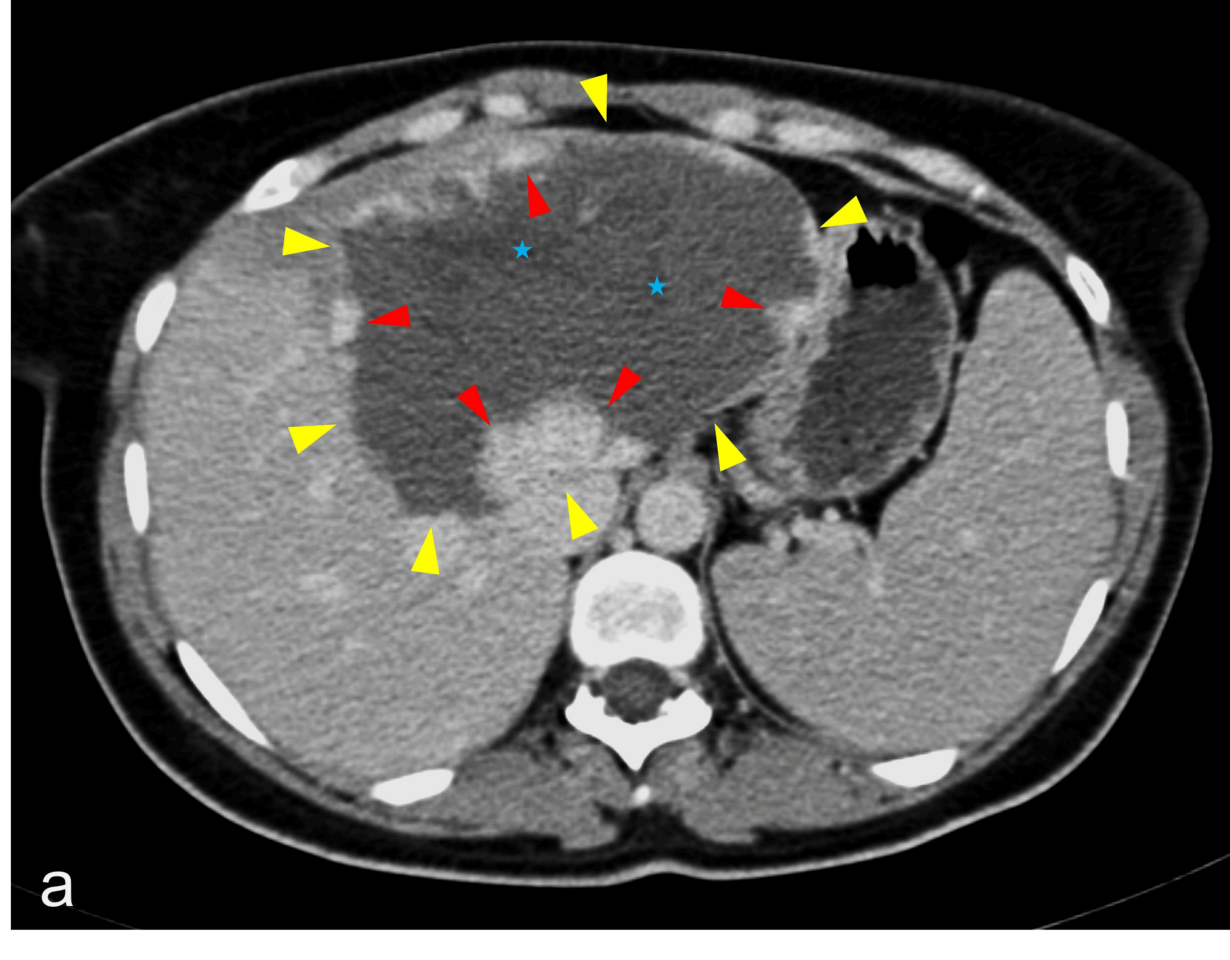
Axial image of intravenous contrast-enhanced CT of the upper abdomen demonstrating a giant cavernous hemangioma occupying the left lateral segment of the liver, with interrupted nodular peripheral enhancement (red arrowheads) and a small central scar (blue star). The lesion margins are delineated by yellow arrowheads.

**Figure 3:**
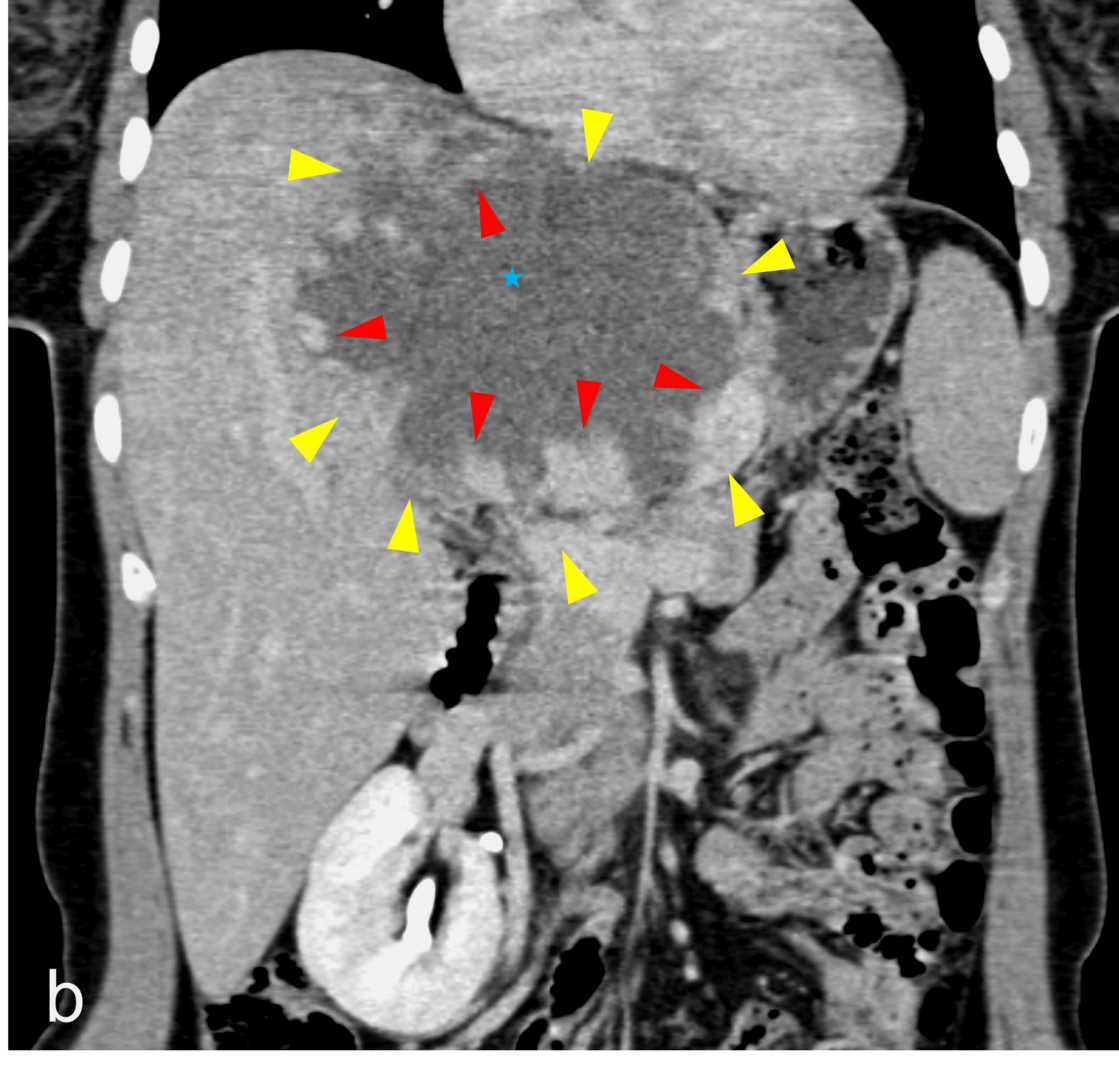
Coronal image of intravenous contrast-enhanced CT of the upper abdomen demonstrating a giant cavernous hemangioma occupying the left lateral segment of the liver, with interrupted nodular peripheral enhancement (red arrowheads) and a small central scar (blue star). The lesion margins are delineated by yellow arrowheads.

Enucleation was avoided in cases with suspected malignancy on preoperative imaging, significant medical comorbidities precluding major abdominal surgery (American Society of Anesthesiologists (ASA) class IV or higher), or when tumor proximity to the primary hilar bifurcation, major hepatic veins, or retrohepatic inferior vena cava was deemed to preclude a pseudocapsular dissection plane. Preoperative imaging assessment of the tumor-IVC interface is essential, particularly for suprahepatic lesions; direct inferior vena cava (IVC) encasement or dense adherence may preclude safe enucleation and should prompt consideration of alternative strategies. Representative preoperative cross-sectional imaging is shown in Figures 2-3.

Description of Surgical Technique

The following technique was applied uniformly across all cases in this series, regardless of tumor location. Technical vigilance and operative planning requirements are heightened for centrally located lesions, as specifically noted where applicable.

Exposure and Setup

**Figure 4:**
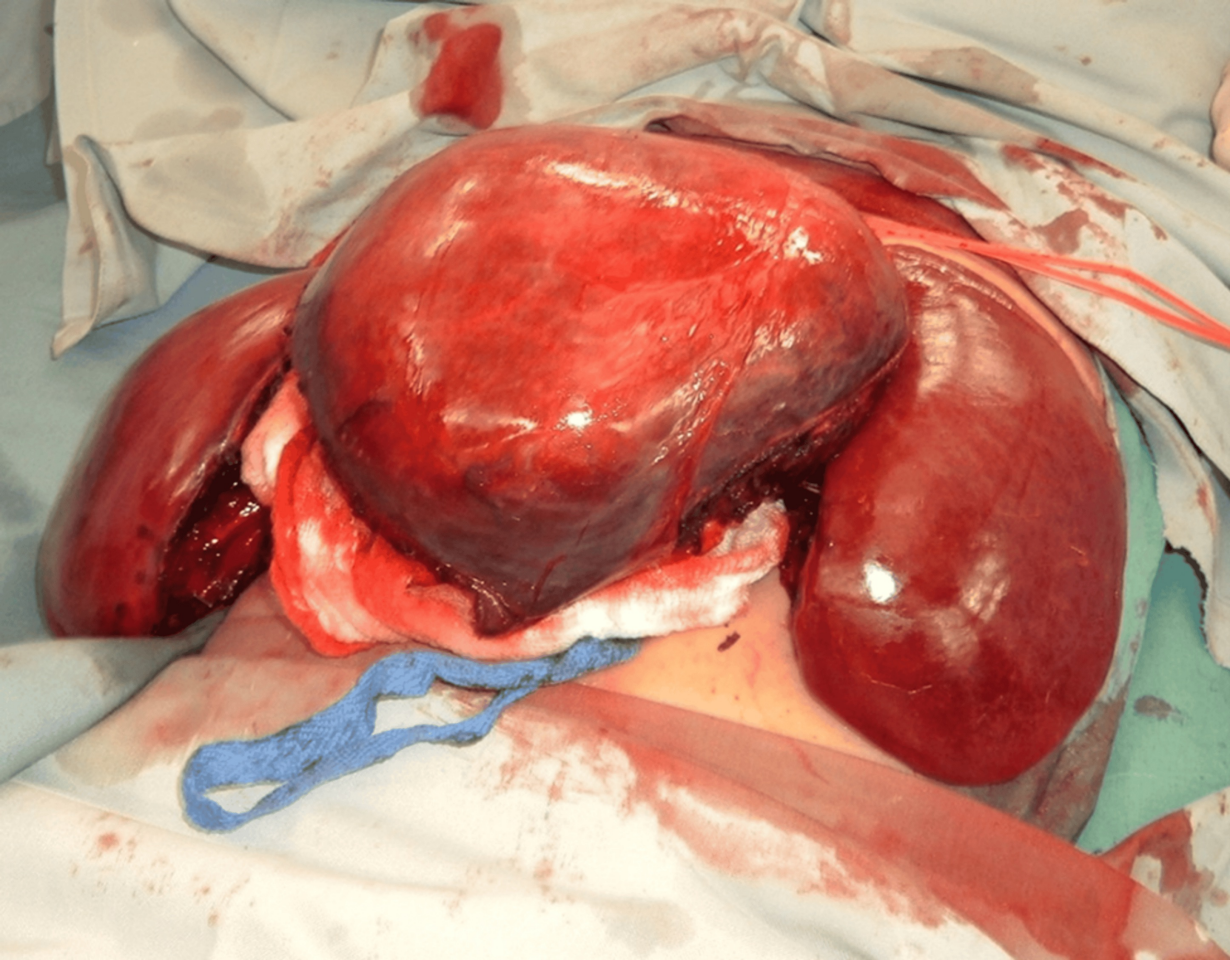
Intraoperative view demonstrating a giant hepatic hemangioma prior to enucleation. The characteristic dark purple-red appearance is evident. Normal liver lobes are visible on both sides of the tumor. A vessel loop is in place for vascular preparation. The lesion's large size and relationship to surrounding structures are clearly appreciable.

A right subcostal incision, extended to the left, as needed, based on tumor location and size, provides wide access to the liver. Thompson's mechanical retractor provides stable, deep exposure. The falciform ligament is divided to facilitate liver mobilization and improve exposure. The hemangioma is typically visualized as a well-defined mass with a characteristic dark purple-red appearance (Figure 4).

IOUS Mapping

IOUS is introduced before any hepatic manipulation using a high-frequency intraoperative linear ultrasound probe. The probe comprehensively examines the hemangioma, delineating its three-dimensional relationship to the left, middle, and right hepatic veins, first-order portal branches, and intrahepatic bile ducts. The thickness and continuity of the pseudocapsule are assessed, typically measuring 2-5 mm at optimal entry sites. Areas where the pseudocapsule appears thickest or where the tumor approaches the capsular surface represent optimal entry points. The surgeon maps the intended dissection plane and identifies potential technical challenges, including proximity to major venous structures or areas of thin pseudocapsule. For centrally located lesions, IOUS mapping is particularly critical given the immediate proximity of the tumor to the middle hepatic vein, hepatocaval confluence, and first-order portal pedicles; all relevant structures must be comprehensively mapped before any incision is made. In cases with suspected IVC proximity, particular attention is paid to characterizing the tumor-IVC interface before proceeding.

Entry into the Pseudocapsular Plane

Based on IOUS guidance, a capsular incision is made over the tumor at the identified optimal entry site. The incision traverses Glisson's capsule and the overlying thin layer of compressed hepatic parenchyma. Gentle blunt dissection with a combination of finger fracture and careful spreading with a right-angle clamp reveals the avascular plane - a distinct interface between the hemangioma's fibrous pseudocapsule and the compressed normal liver parenchyma. Once this plane is established, the natural cleavage is maintained. Entry is deliberate and controlled to avoid capsular rupture, which would result in significant bleeding from the hemangiomatous tissue.

CUSA-Guided Parenchymal Dissection

The CUSA is used to follow the pseudocapsule circumferentially. The CUSA tip is directed parallel to the surface, remaining just at the outer border of the hemangioma, fragmenting and aspirating compressed parenchyma while preserving the fibrous pseudocapsule. Continuous visualization of the pseudocapsule prevents inadvertent penetration into hemangiomatous tissue. The technique provides fine control and excellent tactile feedback, particularly valuable when progressing toward central venous structures. When working near major vessels, lower CUSA power settings and finer tips enhance precision and safety. Any standard ultrasonic aspirator with adjustable power settings is appropriate; the specific system may vary by institutional availability.

Selective Feeder Ligation and Tumor Decompression

**Figure 5:**
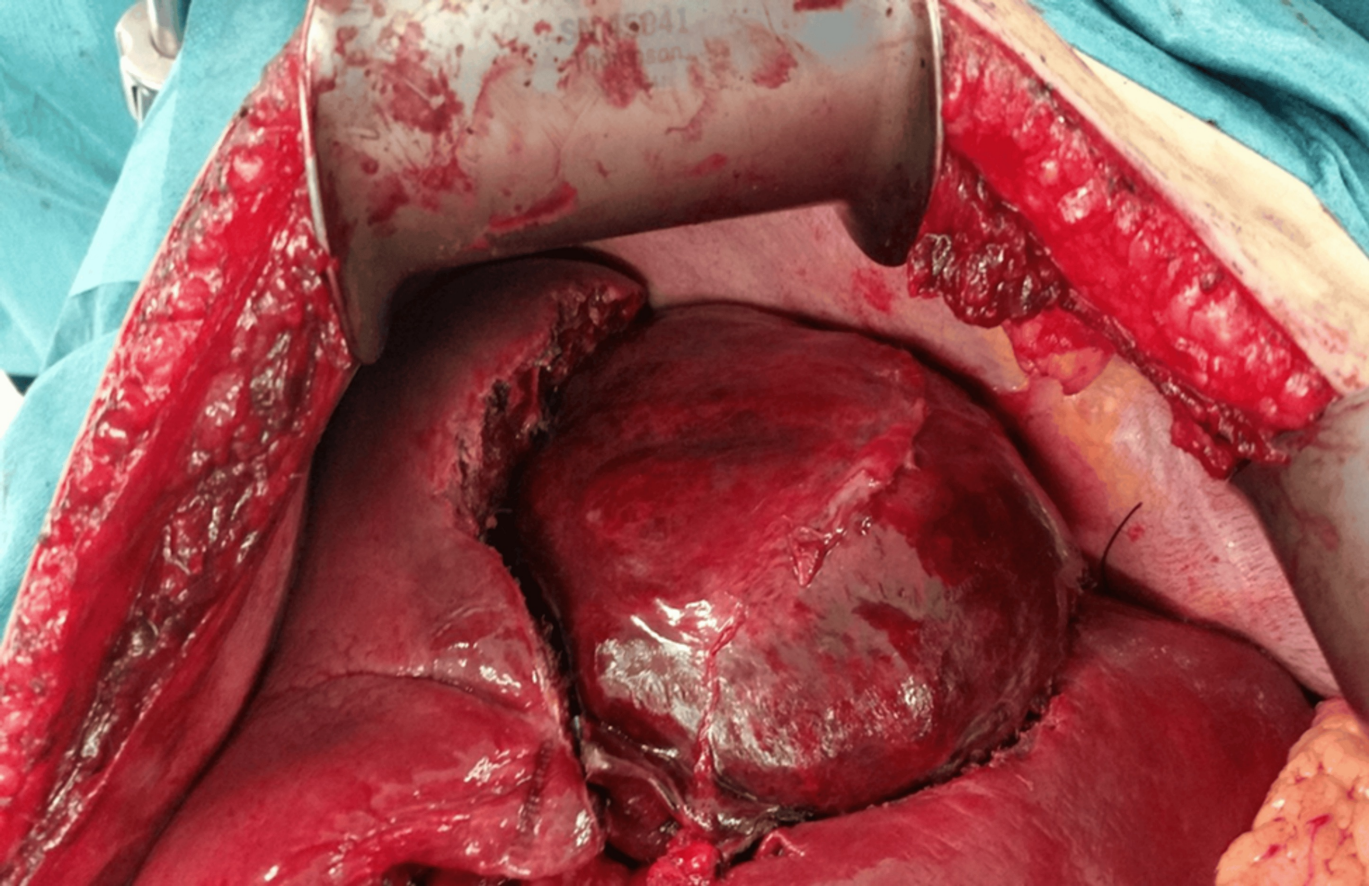
Intraoperative view during pseudocapsular dissection The fibrous pseudocapsule is visible as a distinct white interface between the hemangiomatous tissue and the compressed normal liver parenchyma. The Thompson retractor provides deep surgical exposure.

As the pseudocapsular plane is developed, small arterial and venous feeders entering the hemangioma perpendicular to the dissection plane are encountered. These vessels are individually managed according to their size using suturing, metallic clips, bipolar cautery, or ties before division. Early ligation of arterial feeders, particularly branches from the right or left hepatic artery, produces noticeable tumor decompression. As inflow decreases and feeding vessels are selectively ligated, the hemangioma gradually decreases in size, becomes softer and less turgid, and the pseudocapsule becomes more distinct, improving the dissection plane (Figure 5).

Progressive deflation reduces the risk of capsular rupture during manipulation. Topical hemostatic agents, such as oxidized regenerated cellulose or fibrin-based matrices, are applied as needed at the surgeon's discretion.

Pringle-Free Strategy

A Pringle-free approach is preferred when feasible and was employed in all cases in this series. Maintaining physiologic hepatic perfusion preserves the natural color differential between the dark purple-red hemangiomatous tissue and the pink-brown perfused normal parenchyma, which serves as a continuous visual guide to the correct dissection plane, a particularly valuable orientation aid when operating near the middle hepatic vein or hepatocaval confluence, where anatomic landmarks may be distorted by tumor compression. The avascular nature of the pseudocapsular plane minimizes the bleeding risk when proper technique is employed. The Pringle maneuver was prepared and immediately available in all cases, reserved for scenarios of uncontrolled hemorrhage, capsular rupture, or inadvertent major vessel injury. This Pringle-free strategy is applicable to appropriately selected cases and is not universally applicable to all hemangiomas; readiness for immediate vascular control is essential regardless of lesion location.

Dissection Near Major Venous Structures

As the dissection plane approaches the middle hepatic vein, major portal pedicles, or hepatocaval confluence, IOUS is repeated to reassess the anatomic relationship and confirm the dissection trajectory. This phase requires meticulous technique and represents the most technically demanding aspect of central hemangioma enucleation. Dissection proceeds in millimeter increments using fine CUSA tips, precise bipolar cautery for small vessels, and gentle retraction. When the pseudocapsule thins to less than 2-3 mm near major veins--a common occurrence in central lesions--sharp microdissection with fine scissors or a scalpel may be employed. Small venous tributaries are carefully identified and ligated with fine sutures or clips before division. Patience is paramount; this phase often consumes a disproportionate percentage of total operative time despite representing a small fraction of the dissection circumference.

Final Separation and Assessment

**Figure 6:**
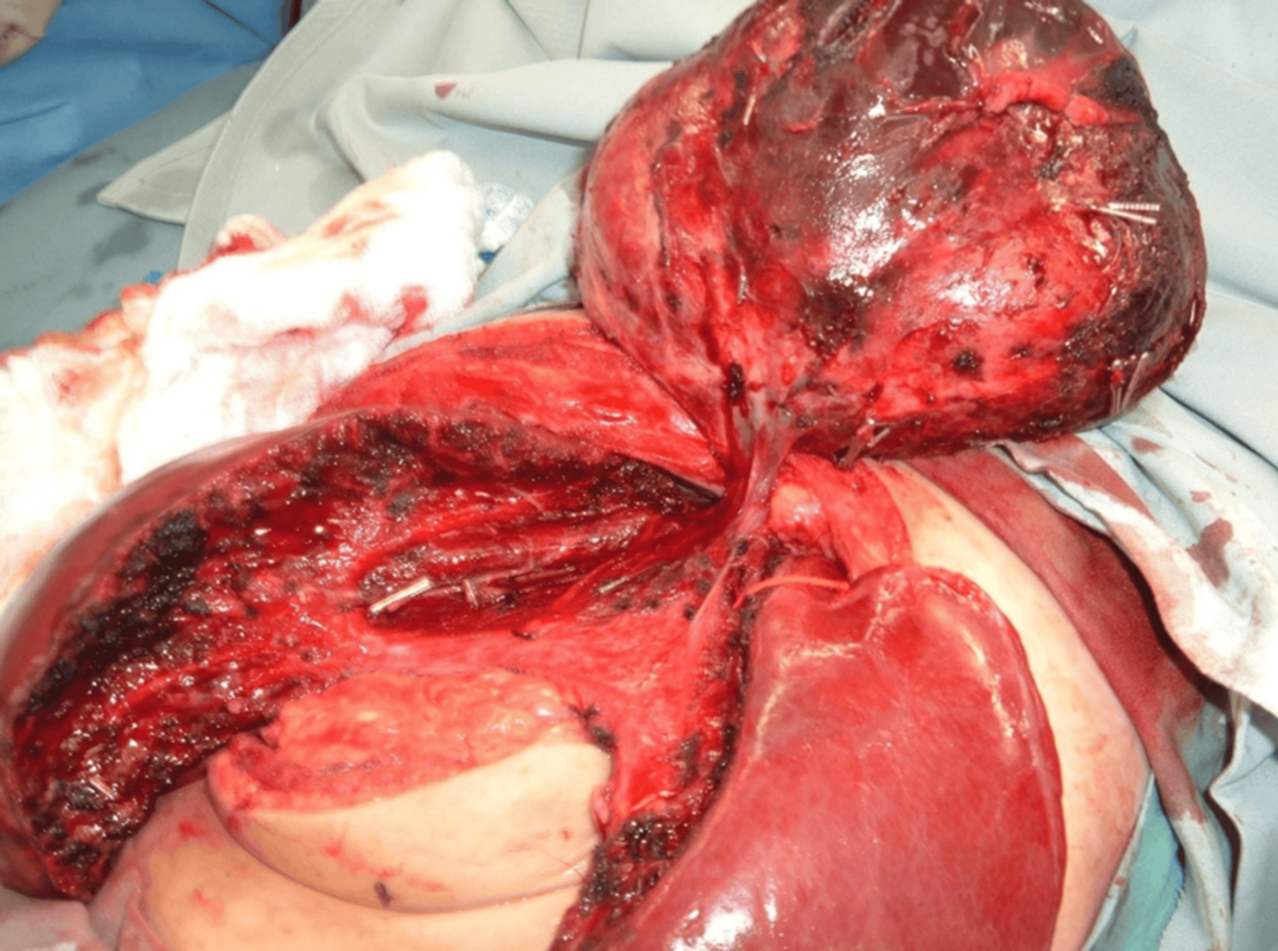
Intraoperative view during CUSA-guided enucleation demonstrating selective feeder ligation Multiple metallic clips are visible on individually ligated tumor-feeding vessels. The progressive dissection reveals the tumor's relationship to the surrounding liver parenchyma. CUSA: Cavitron Ultrasonic Surgical Aspirator

Once the circumferential dissection is complete, the lesion is gently delivered from the hepatic cavity. The raw hepatic surface is thoroughly examined for active bleeding, venous oozing, and bile leakage. Minor oozing is managed with topical hemostatic agents and selective bipolar cauterization. Bile leaks are identified using saline irrigation and addressed with direct suture repair. IOUS may be repeated to confirm preserved flow in adjacent major hepatic veins. One or two closed suction drains are placed adjacent to the resection cavity; drain placement was performed routinely in all six cases in this series, regardless of intraoperative findings, and was maintained until output was serous and below 50 mL per day (Figure 6).

**Figure 7:**
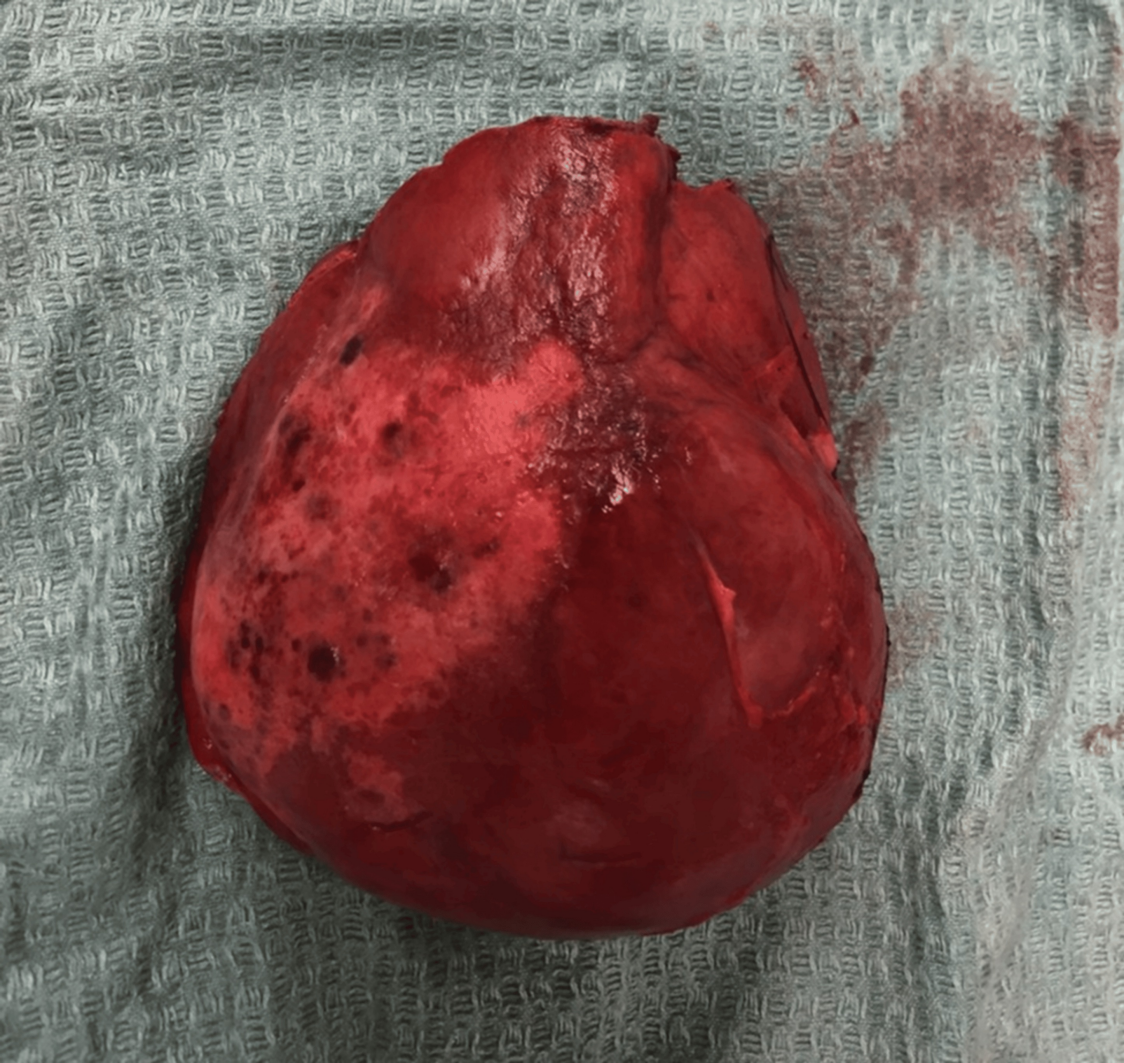
Excised hemangioma specimen demonstrating the well-circumscribed lesion with intact pseudocapsule The characteristic dark red-purple hemorrhagic surface with areas of fibrous pseudocapsule tissue is clearly visible.

The excised specimen reveals a well-circumscribed lesion with an intact pseudocapsule (Figure 7).

Technical Tips and Key Considerations

Intraoperative ultrasound utilization: Intraoperative ultrasound is used at three key stages: before incision for entry point selection and pseudocapsule mapping; during dissection when approaching major vascular structures; and following tumor removal to confirm preserved flow within adjacent major hepatic veins and portal structures. For peripheral lesions, IOUS provides useful guidance for entry point selection. For central lesions, IOUS is indispensable and should be repeated at any stage where anatomic uncertainty arises.

Pseudocapsular plane management: Dissection is initiated at locations where the pseudocapsule is thickest or closest to the liver surface. Strict adherence to the pseudocapsular plane is maintained throughout, as deviation into normal hepatic parenchyma increases the risk of bleeding and bile leakage. Once the plane is established, parenchymal dissection is preferentially performed using the CUSA rather than blunt techniques, with sharp dissection reserved for areas where the pseudocapsule becomes thin near major vascular structures. In the event of capsular rupture, temporary hemostasis is achieved with packing, followed by direct repair of the capsule using a running suture before continuation of the dissection.

Vascular control: Early ligation of arterial feeders is emphasized to promote progressive tumor decompression and improve visualization of the pseudocapsular plane. Clips or suture ligation are preferred over energy devices when operating near major vascular structures to minimize thermal injury. Traction near the central hepatic veins is reduced to prevent avulsion injuries. If a major venous injury occurs, proximal and distal vascular control is obtained prior to attempted repair. One case in this series involved direct IVC adherence resulting in a laceration managed with primary suture repair, underscoring the importance of preoperative imaging assessment of caval involvement as a patient selection criterion.

Hemostasis and bile leak management: Following tumor removal, the raw hepatic surface is carefully inspected for active bleeding, venous oozing, or bile leakage. Saline irrigation is used to identify bile leaks. Persistent oozing is managed with topical hemostatic agents rather than excessive cauterization. For large raw surfaces, omental coverage may be employed for additional protection and hemostasis support.

Decision-making for enucleation versus conversion to resection: Enucleation is pursued when a clearly identifiable pseudocapsular plane is present, major vascular structures are compressed rather than encased by the tumor, and visualization remains controlled. Conversion to formal hepatic resection is considered when the pseudocapsular plane becomes indistinct or absent, major vascular encasement rather than compression is encountered, uncontrolled hemorrhage persists despite temporary inflow occlusion, multiple capsular ruptures occur, suggesting invasive behavior, or atypical imaging features raise concern for an alternative diagnosis.

Postoperative management:* *Patients are monitored in a general surgical ward unless intraoperative complications warrant intensive care. Liver function tests and hemoglobin levels are checked on postoperative day one and as clinically indicated. Drains are maintained until output decreases below 50 mL per day with serous character, typically removed by postoperative day 3 to 5. Routine postoperative imaging is not required in uncomplicated cases. Patients are discharged on postoperative day 4 to 7, depending on clinical recovery.

Results

Patient Characteristics

Six consecutive patients underwent open enucleation of giant hepatic hemangiomas at AUBMC over an eight-year period using the described technique. All procedures were performed by surgeons with extensive prior hepatobiliary experience at a high-volume tertiary center, with a well-established institutional practice in open liver surgery, including hepatic resections and enucleations performed prior to the study period. Patient ages ranged from 26 to 50 years (median 40 years), and five of six patients were female (83%), consistent with the known female predominance of hepatic hemangiomas. One patient was male. Two patients had documented hormonal exposure histories: one with prior oral contraceptive use and one with a prior cesarean delivery. Patient and tumor characteristics are detailed in Table 1.

**Table 1:** Patient characteristics and operative outcomes *Preoperative MRI confirmed giant hemangioma — exact dimensions not available in accessible records; surgical specimen 11.5x10x5 cm. †Giant hemangioma confirmed on triphasic CT at outside institution prior to referral — exact dimensions not available. §Case 4 presented with two synchronous hemangiomas: one in the left hepatic lobe (segments VI/VII, 10.5x9x9.9 cm) and one in the right lobe (7x3.3 cm), both resected in the same operative setting. CD = Clavien-Dindo grade; CUSA = Cavitron Ultrasonic Surgical Aspirator; EBL = Estimated blood loss; F = Female; IVC = Inferior vena cava; LOS = Length of stay; M = Male; ND = Not documented in available records; N/A = Not applicable Preoperative tumor size was measured from the most recent available cross-sectional imaging study (CT or MRI) prior to surgery. Formal cross-modality size comparison was not performed, given the retrospective design.

	Case 1	Case 2	Case 3	Case 4	Case 5	Case 6
Patient	Patient 1	Patient 2	Patient 3	Patient 4	Patient 5	Patient 6
Age (years)	50	43	33	42	26	36
Sex	F	F	M	F	F	F
Segments involved	IV	V/VI/VII/VIII	IV/V	VI/VII	IV/V/VI	Suprahepatic/IVC
Location category	Central	Mixed	Central	Peripheral	Mixed	Complex
Preop size (cm)	12.3x10x8.1	Giant*	8x6.7	10.5x9x9.9 (Lt) 7x3.3 (Rt)	12.9x11.4x13.5	Giant†
Pathology size (cm)	ND	11.5x10x5	6.5x6.5x3	13x10x6 (Lt) 6.5x5x2.5 (Rt)	14.5x13x7.5	ND
Surgical indication	Symptomatic	Growth + symptoms	Symptoms + diagnostic uncertainty	Symptomatic (2 synchronous lesions)§	Growth + symptoms	Symptomatic + pressure
Hormonal history	ND	ND	N/A (male)	OCP history	Prior C-section	ND
EBL (mL)	150	ND	ND	ND	500	~500
Transfusion	None	None	None	None	None	3 units
LOS (days)	5	7	10	7	~7	7
Pringle maneuver	No	No	No	No	No	No
CUSA used	Yes	Yes	Yes	Yes	Yes	Yes
Drain placed	Blake drain	Blake drain	Blake drain	ND	Closed suction	Blake drain
Complications	None	None	None	Urinary retention (CD-I)	None	IVC laceration (CD-III)
Pathology confirmed	Cavernous hemangioma	Cavernous hemangioma	Cavernous hemangioma	Cavernous hemangioma	Cavernous hemangioma	Cavernous hemangioma

Of the six cases, two involved purely central hepatic segments (IV and IV/V, respectively), three involved mixed central and peripheral segments (V/VI/VII/VIII, IV/V/VI, and V/VI), and one involved a complex suprahepatic location with direct inferior vena cava adherence. Preoperative tumor sizes ranged from 7 to 12.9 cm on cross-sectional imaging, confirming giant hemangioma in all cases meeting the adopted threshold of ≥5 cm. Surgical indications included symptomatic presentation in four cases, progressive enlargement with symptoms in two cases, and diagnostic uncertainty in one case (central hemangioma with obstructive jaundice raising concern for malignancy). In the two cases where progressive enlargement was the operative indication, interval growth was documented on at least two cross-sectional imaging studies performed 6-12 months apart and was considered clinically meaningful by the treating surgeons and multidisciplinary team, though exact measurement differentials were not uniformly available for retrospective extraction. None of the six patients presented with consumptive coagulopathy or Kasabach-Merritt syndrome. One case presented with two synchronous hemangiomas in the left and right hepatic lobes.

Operative Outcomes

All six cases were completed without conversion to formal anatomical hepatectomy. CUSA-guided pseudocapsular dissection was employed in all cases, and the Pringle maneuver was not required in any case. Mean operative time was approximately 3 hours (range 2-4 hours). Documented estimated blood loss ranged from 150 to 500 mL in Cases 1, 5, and 6, the 3 cases for which intraoperative anesthesia records were available; the remaining 3 cases (Cases 2, 3, and 4) had no transfusion requirement and no documented EBL in available records, consistent with similarly low intraoperative blood loss. No bile leaks, postoperative liver failure, or mortality occurred. Length of hospital stay ranged from 5 to 10 days (median 7 days). All pathology specimens confirmed the diagnosis of cavernous hemangioma.

One case (Case 6) was complicated by an intraoperative inferior vena cava laceration due to direct tumor adherence to the caval wall, which was managed with primary suture repair requiring three units of packed red blood cells (Clavien-Dindo grade III). This represents the only transfusion requirement in the series. The patient recovered uneventfully with a hospital stay of seven days. One additional patient (Case 4) experienced urinary retention requiring Foley catheter reinsertion (Clavien-Dindo grade I), which resolved without further intervention. The remaining four cases were completed without any complications. All patients were followed clinically at 1 and 3 months postoperatively per standard institutional protocol; no routine cross-sectional imaging was performed in the absence of symptoms, consistent with current practice for confirmed benign lesions. No disease recurrence, late complications, or hemangioma-related readmissions were identified during the follow-up period.

## Discussion

Technical considerations

A key observation of this technical report is that the core pseudocapsular enucleation technique, CUSA-guided dissection, selective feeder ligation, and Pringle-free inflow strategy, was applied uniformly and successfully across all six cases, regardless of tumor location. This supports the generalizability of the described technique and suggests that the fundamental operative principles of pseudocapsular enucleation are applicable to giant hepatic hemangiomas in any hepatic segment. What differs between central and peripheral lesions is not the technique itself but the level of technical vigilance required: centrally located lesions demand heightened attention to IOUS-guided trajectory planning, meticulous millimeter-increment dissection near major hepatic veins, and structured decision algorithms for recognizing and managing proximity to first-order portal structures and the hepatocaval confluence [4,5]. In cases presenting with atypical imaging features, multidisciplinary review was undertaken prior to proceeding with enucleation, and specimens retrospectively confirmed cavernous hemangioma, supporting the validity of the described selection criteria.

While laparoscopic approaches have demonstrated advantages for peripheral hepatic hemangiomas, recent evidence highlights the continued relevance of open enucleation for centrally located lesions. Li et al. reported that laparoscopic enucleation of hemangiomas in special hepatic segments (I, IVa, VII, VIII) was associated with significantly higher intraoperative blood loss, elevated blood transfusion rates, and a numerically higher conversion rate compared with normal locations, often necessitated by injuries to major vessels [4]. These findings underscore the technical challenges of managing central hemangiomas through a minimally invasive approach and highlight the importance of careful patient selection and detailed operative guidance.

Open enucleation offers specific advantages for central giant hemangiomas. Direct surgical access provides immediate vascular control through manual compression or suturing in the event of major hemorrhage, critical given the proximity of these tumors to major hepatic veins and the inferior vena cava. The tactile feedback available during open surgery facilitates precise identification and maintenance of the pseudocapsular plane, which is essential for parenchymal preservation in compressed central anatomy.

The deliberate avoidance of the Pringle maneuver represents a key technical consideration applicable to both central and peripheral enucleation. Published series report variable use of inflow occlusion during hemangioma enucleation, with several centers employing intermittent Pringle as a default strategy, particularly in the laparoscopic setting [6,8,9]; reported intraoperative transfusion rates across published enucleation series range widely, reflecting heterogeneous case complexity and inflow occlusion strategies. Beyond hemostatic considerations, the Pringle-free approach provides a critical navigational advantage in central anatomy: preserved perfusion maintains the natural color differential between the dark purple-red hemangiomatous tissue and the pink-brown normal parenchyma, serving as a continuous real-time guide to the dissection plane at the hepatocaval confluence and near the middle hepatic vein, anatomic zones where ischemic color change from inflow occlusion would eliminate this visual landmark entirely. This navigational benefit, not simply blood loss reduction, represents the primary rationale for the Pringle-free strategy in centrally located lesions and has not been systematically emphasized in prior technical descriptions.

Hormonal and obstetric considerations

The female predominance observed in our series (83%, median age 40 years) is consistent with the well-established epidemiology of giant hepatic hemangiomas and reflects the patient population surgeons will most commonly encounter in clinical practice. Given that a substantial proportion of patients presenting for surgical management are women of reproductive age, practical guidance on hormonal and obstetric considerations is a clinically relevant component of the preoperative and postoperative counseling framework for this condition, irrespective of whether individual cases have documented hormonal exposure histories. The hormonal responsiveness of hepatic hemangiomas has important implications for this population. In a prospective cohort study, Glinkova et al. demonstrated higher rates of lesion enlargement among women exposed to exogenous estrogen [12]. Additional observational data describe interval growth during pregnancy [13]. For women with large lesions (>5-10 cm), particularly those contemplating pregnancy, baseline imaging with selective interval surveillance during gestation may be considered. Routine discontinuation of oral contraceptives or hormone replacement therapy is not universally recommended; rather, shared decision-making is appropriate, especially in patients demonstrating documented growth [12]. Early referral to hepatobiliary surgery may be reasonable in cases of progressive enlargement or compressive symptoms, permitting elective management before pregnancy when feasible.

Limitations

This report has limitations that are inherent to and consistent with its intent as a technical educational report rather than a comparative outcomes study. As with all technical reports, the series is intentionally focused rather than powered for statistical inference; the primary aim is operative reproducibility and structured guidance, not outcome comparison across surgical approaches. All procedures were performed by surgeons within the same hepatobiliary unit at AUBMC, ensuring a consistent operative philosophy, shared technical training, and institutional familiarity with the described approach across all cases, which supports the technical consistency of the reported experience. Granular data, including serial imaging measurements, specific preoperative imaging modalities, and operative blood loss, were not uniformly available across all cases, given the retrospective design and eight-year study period, which is consistent with the descriptive intent of the report and does not affect the validity or reproducibility of the described technique.

The described technique reflects enduring operative principles that remain directly applicable to contemporary hepatobiliary practice, with technical steps, instrumentation, and decision algorithms consistent with current standards. Patients were followed clinically per institutional protocol with no complications or recurrence identified, supporting the safety of the approach within the available follow-up period. The intent of this report is not to establish superiority over alternative approaches but to provide detailed, reproducible operative guidance for a challenging scenario that remains underrepresented in the surgical literature. Prospective multicenter validation in larger series would be a valuable next step to further establish generalizability across institutions and surgeons.

## Conclusions

This technical report provides detailed operative guidance for open parenchyma-preserving enucleation of giant hepatic hemangiomas across central, peripheral, and complex tumor locations, illustrated through a retrospective consecutive series of six cases at a single tertiary institution. The described CUSA-guided pseudocapsular technique without inflow occlusion was applied uniformly and successfully across all anatomical locations, underscoring its reproducibility and adaptability. Technical emphasis is placed on centrally located lesions involving segments IV, V, and VIII, where proximity to major hepatic veins and portal structures demands structured operative planning, heightened IOUS utilization, and readiness for immediate vascular control. This report complements existing laparoscopic literature by providing systematic open operative guidance for the anatomically challenging cases where open enucleation remains the safest and most appropriate strategy.
